# Drug-associated porphyria: a pharmacovigilance study

**DOI:** 10.1186/s13023-024-03294-8

**Published:** 2024-08-01

**Authors:** Qi Wang, Jun ling Zhuang, Bing Han, Miao Chen, Bin Zhao

**Affiliations:** 1 Department of Hematology, Peking Union Medical College Hospital (PUMCH), Chinese Academy of Medical Sciences and Peking Union Medical College, Beijing, China; 2 Department of Pharmacy, Peking Union Medical College Hospital (PUMCH), Chinese Academy of Medical Sciences and Peking Union Medical College, Beijing, China

**Keywords:** Porphyria, Drug-induced porphyria, FAERS, Drug porphyrogenicity

## Abstract

**Background:**

The potentially fatal attacks experienced by porphyria carriers are triggered by various porphyrinogenic drugs. However, determining the safety of particular drugs is challenging.

**Methods:**

We retrospectively used the U.S. Food and Drug Administration’s Adverse Event Reporting System (FAERS) to identify drugs associated with porphyria as an adverse event (AE) extracted from data from January 2004 to March 2022. The associated search terms included “Porphyria,” “Porphyria screen,” “Porphyria non-acute,” “Porphyria acute,” “Acquired porphyria,” and “Pseudoporphyria.” Signal mining analysis was performed to identify the association between drugs and AEs by four algorithms, namely the reporting odds ratio, proportional reporting ratio, Bayesian confidence propagation neural network, and multi-item gamma Poisson shrinker.

**Results:**

FAERS reported 1470 cases of porphyria-related AEs, and 406 drugs were screened after combining trade and generic names. All four algorithms identified 52 drugs with signals. The characteristics of all the reports and signaling drugs were analyzed.

**Conclusions:**

This is the first report of drug-associated porphyria that provides critical information on drug porphyrogenicity, facilitating rational and evidence-based drug prescription and improving the accuracy of porphyrogenicity prediction based on model algorithms. Moreover, this study serves a reference for clinicians to ensure that porphyrinogenic drugs are not prescribed to carriers of porphyria genetic mutations.

## Background

Porphyria is a group of rare metabolic diseases caused by inherited or acquired enzymatic deficiency in the metabolic pathway of heme biosynthesis [1]. Eukaryotic heme biosynthesis comprises eight enzymatic reactions; each type of porphyria is associated with a different defect. The first and rate-limiting step in the heme biosynthetic pathway involves the formation of δ-aminolevulinic acid (ALA) from the condensation of glycine and succinyl CoA by δ-aminolevulinic acid synthase (ALAS)1 in the liver and ALAS2 in the erythroblastic system [2]. Porphyria is classified as hepatic or erythroid, depending on whether the excess production of porphyrin precursors or porphyrins arises from and accumulates in the liver or developing erythrocytes, respectively. It is also classified clinically as acute or cutaneous based on the respective major clinical manifestations [3]. Acute hepatic porphyria (AHP) includes acute intermittent porphyria (AIP), hereditary coproporphyria (HCP), variegate porphyria (VP), and δ-aminolevulinic acid dehydratase deficient porphyria (ADP). All four types of AHP present with acute abdominal and neuropsychiatric symptoms. For example, porphyria cutanea tarda (PCT) is a hepatocutaneous porphyria, as it presents with cutaneous lesions, but the primary site of porphyrin accumulation is the liver. Meanwhile, erythropoietic porphyria includes congenital erythropoietic porphyria (CEP), erythropoietic protoporphyria (EPP), and X-linked protoporphyria (XLP), and presents with cutaneous photosensitivity [4]. AIP is clinically the most common type of acute porphyria, with a prevalence of 1 carrier per 2000 persons in Western populations [5]. However, acute attacks occur in less than 10% of the at-risk population [6]. HCP is markedly less prevalent than AIP, with HCP and ADP considered very rare with no reliable epidemiological data available. Acquired PCT is the most prevalent cutaneous porphyria, estimated to affect 5–10 persons per 100,000 population [7]. Porphyria diagnosis is made based on corresponding clinical manifestations and significantly elevated laboratory indexes, such as urinary heme precursors (PBG and ALA) and urinary, fecal, erythrocyte and plasma porphyrins. Enzyme activity measurement and genetic testing are recommended to confirm the type of porphyria and help identify asymptomatic carriers.

The potentially fatal attacks experienced by patients with AHP are triggered by various porphyrinogenic factors, including starvation, infection, alcohol consumption, menstruation, and certain commonly prescribed drugs, including cytochrome P450 (CYP450)-inducing agents [5]. Some drugs also precipitate PCT [6], including barbiturates, estrogen, griseofulvin, rifampicin, sulfonamides, and nitrofurantoin [7]. Porphyrinogenic drugs induce the hepatic expression of *ALAS1* or enhance utilization and depletion of hepatic regulatory heme, producing more neurotoxic porphyrin precursors, ALA, and porphobilinogen (PBG) [8]. It is, therefore, important for medical providers to accurately determine the safety of drugs for use in carriers of porphyria genetic mutations. Clinical case reports of drug side effects and analysis of drug structural and functional information [9], as well as experimental systems using animal or cell culture models, have been used to predict the porphyrogenicity of drugs. However, minute changes in the chemical structure of porphyrinogenic drugs may essentially modify their effect, and in vitro models differ greatly from human physiology, impeding the determination of a specific drug’s safety. Hence, a resource compiling the drugs that induce porphyria in real-world practice will serve to not only verify the porphyrogenicity of drugs but also provide valuable information regarding safe drug use in porphyria.

The Food and Drug Association’s Adverse Event Reporting System (FAERS) is a post-marketing surveillance program seeking voluntary input on adverse drug reactions (ADRs) to monitor drug safety; as such, it is the world’s largest repository of reported hazardous drug events [10]. Although adverse events (AEs) are reported to the FDA by healthcare professionals, consumers, and manufacturers [11], research gaps exist in the study of drugs that induce porphyria in clinical practice using big data. Accordingly, in this study, we conducted a retrospective pharmacovigilance study using data from the FAERS database to perform signal mining analysis and extract drugs with AEs related to porphyria. We then presented the characteristics of drugs inducing porphyria in clinical practice using big data.

## Methods

### Data source collection

We used FAERS data covering the from January 2004 to March 2022. AEs were identified using the MedDRA terms “Porphyria (10036181),” “Porphyria screen (10050928),” “Porphyria non-acute (10036182),” “Porphyria acute (10036182),” “Acquired porphyria (10053147),” and “Pseudoporphyria (10037145).” Drugs in the FAERS database can be registered using different conventions. Report listings include the primary suspect (PS) or secondary suspect (SS) agent. Herein, we selected the PS of the porphyria drugs, and 1470 drugs were obtained.

### Data mining

The whole process can be divided into the following four steps:

Step 1 In the FAERS database, we first used “adverse events” as a search condition and “Porphyria” as a search condition; we selected the PS of the porphyria drugs to obtain the corresponding 1470 drug names.
Step 2 Since generic names and trade names are both reported in the FAERS, we manually used www.drugbank.com to match the generic and trade names of each drug; ultimately, 406 unique drugs were identified.
Step 3 In the US FDA’s spontaneous reporting database, drug-event combinations are disproportionately present; hence, screening algorithms and computer systems are used to effectively signal higher-than-expected combinations of drugs and events. Signaling drugs are associated with the occurrence of corresponding adverse events, as defined by the Adverse Reactions Database Reporting System.

**Fig. 1:**
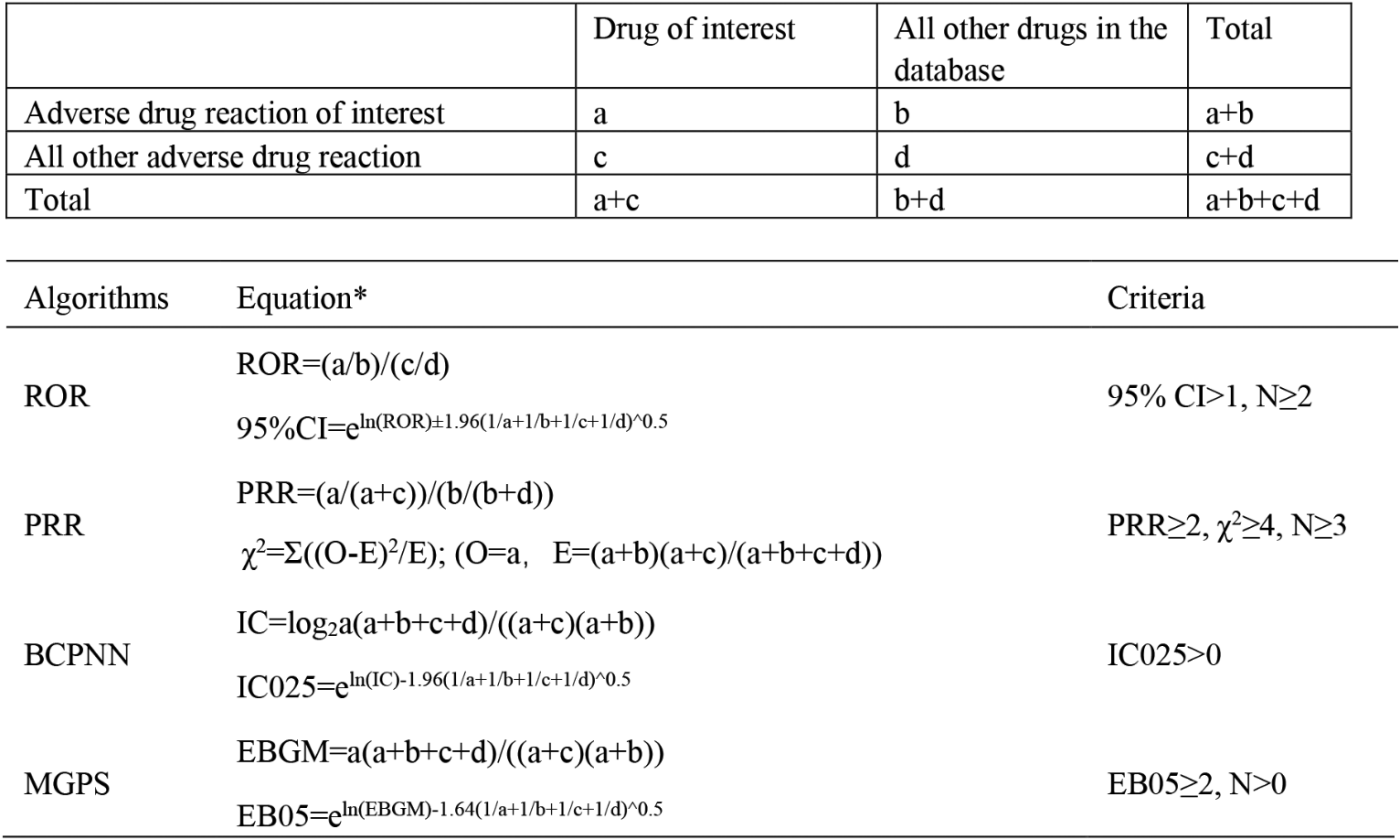
Specific contents of the four algorithms

Four screening algorithms, namely the reporting odds ratio (ROR), proportional reporting ratio (PRR), Bayesian confidence propagation neural network (BCPNN), and multi-item gamma Poisson shrinker (MGPS) [12–20], were used to identify the association between drugs and AEs. Additionally, these four algorithms were employed to calculate the corresponding values for each drug. The specific contents of the four algorithms are shown in Fig. 1.

**Fig. 2:**
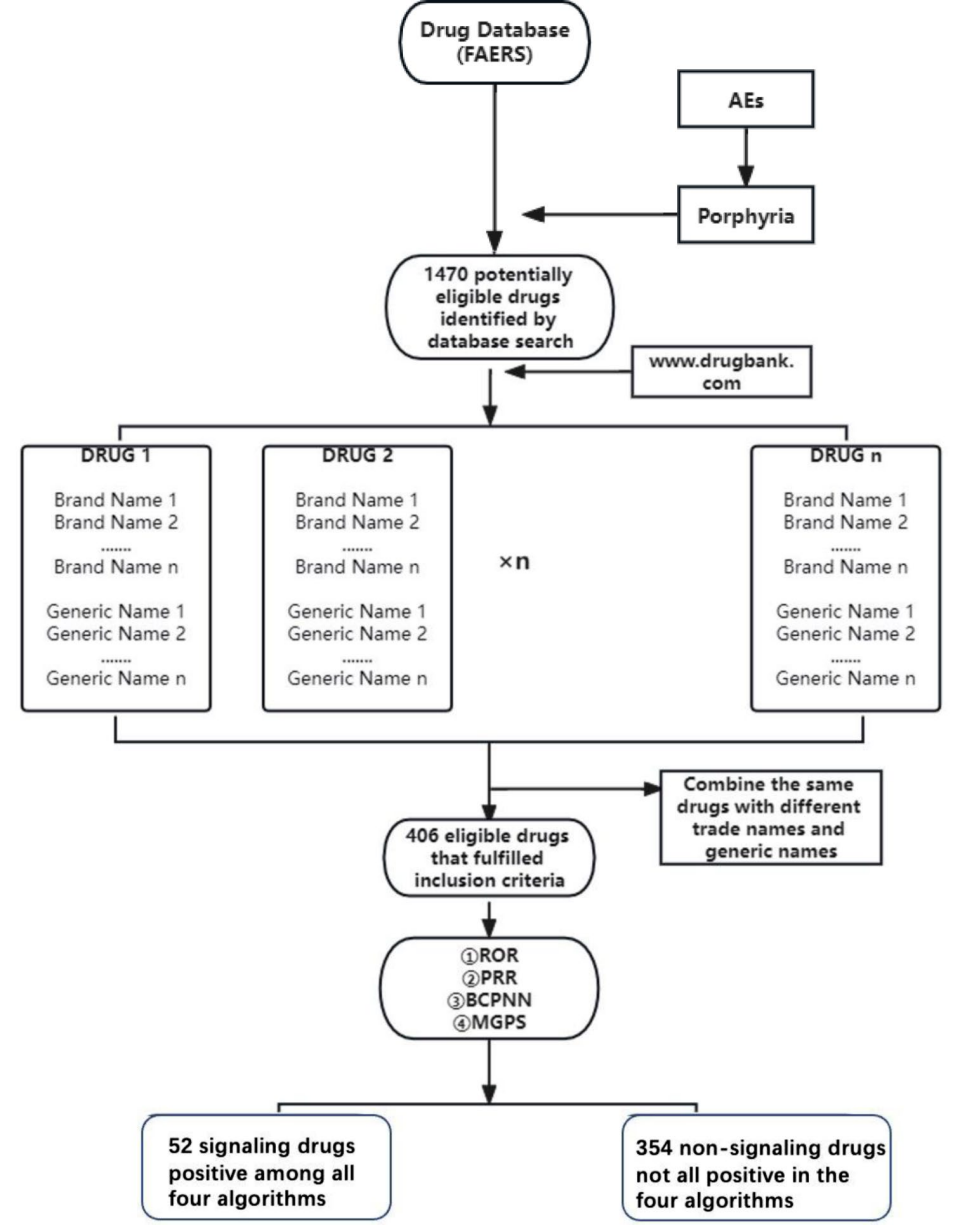
Flowchart of drugs associated with porphyria obtained from the FAERS database

Step 4: Drugs were classified as signaling/non-signaling according to whether the drug values were positive among all four algorithms. The flowchart is depicted in Fig. 2.

### Signal detection

In contrast to clinical trials, where the incidence of adverse reaction is easily computed, counts of spontaneously reported drug-event combinations cannot be assessed as “large” or “small” without a comparison value. Therefore, we defined a drug with a positive result in all four algorithms as having “signals,” defined in CIOMS VI as “a report or reports of an event with an unknown causal relationship to treatment that is recognized as worthy of further exploration and continued surveillance” [21]. By detecting signals, 52 signaling drugs were obtained; all were associated with porphyria and signal detection was positive among all four methods.

### Production of the data analysis table

The anatomical therapeutic chemical (ATC) system is a global standard overseen by the World Health Organization (WHO) [22], that allocates each drug a unique code using the ATC classification system. This alpha-numeric code begins with a letter that represents a system of the human body. The ATC codes were applied to classify the 52 signaling drugs into nine categories, namely, J-general anti-infectives drugs, L-antineoplastics and immunomodulating agents drugs, A-alimentary tract and metabolism drugs, N-central nervous system drugs, C-cardiovascular drugs, G-genitourinary and sex hormones drugs, D-dermatological drugs, B-blood and blood-forming organ drugs, M-musculoskeletal drugs.

## Results

### Characteristics of the FAERS study population

#### Data characteristics

In total, 406 drugs from 1470 cases reported between January 2004 and March 2022 were included in the analysis for porphyria-related drugs from the FAERS. A clear relationship was observed between drug use and adverse drug reactions. The characteristics of the patients from the FAERS are presented in Table 1.

**Table 1:** Demographic characteristics of patients from the FAERS

Characteristic	Value
Number of patients, n	1470
Age, years	
< 18 years old, *n* (%)	94 (6.39%)
18–44 years old, *n* (%)	396 (26.94%)
45–64 years old, *n* (%)	325 (22.11%)
> 64 years old, *n* (%)	227 (15.44%)
Unknown, *n* (%)	428 (29.12%)
Sex	
Male, *n* (%)	573 (38.98%)
Female, *n* (%)	692 (47.07%)
Unknown, *n* (%)	205 (13.95%)
Outcome events	
Death, *n* (%)	54 (3.31%)
Disability, *n* (%)	35 (2.03%)
Hospitalization, *n* (%)	535 (31.01%)
Life-threatening condition, *n* (%)	45 (2.61%)
Need for further intervention, *n* (%)	3 (0.17%)
Other adverse events, *n* (%)	1033 (59.88%)
Unknown, *n* (%)	20 (1.12%)
Distribution of reporters	
Health-care professionals, *n* (%)	964 (69.80%)
Consumers, *n* (%)	240 (16.32%)
Unknown, *n* (%)	204 (13.89%)

#### Reporting country distribution

The top three countries by number of reports for porphyria were the United States of America, France, and Spain (38.71%, 8.78%, and 5.31%, respectively).

#### Age and sex

The most common reporting age range was 18–44 years old (26.94%), followed by 45–64 years old (22.11%). More cases involved females than males (47.07% vs. 38.98%, respectively).

#### Distribution of reporters

The FARES database contains AEs submitted by healthcare professionals, such as doctors, nurses, and pharmacists, and consumers, who may include patients, family members, or lawyers. Of the 1470 cases, most were submitted by healthcare professionals (69.80%), followed by consumers (13.79%), while 13.88% of reporters did not include their identifying information.

#### Outcome events and incidence

Through the FAERS system, we obtained a total of seven adverse outcomes. We determined the associated prognosis of porphyria after using these drugs based on the outcomes: death (3.31%), disability (2.03%), hospitalization (31.01%), life-threatening condition (2.61%), need for further intervention to prevent permanent impairment/damage (0.17%), and other adverse events (59.88%).

### Characteristics of signaling drugs

The corresponding 52 drugs were identified as signal-positive using the four methods through the four algorithms.

#### Adverse reaction onset-time

Generally, the most common time to onset of drug-associated porphyria was within 1 month (106; 39.70%) of medication prescription, the second was more than 1 year (65; 23.34%), followed by within 3 months to 1 year (49; 18.35%) and within 2–3 months (47; 17.60%).

#### Drug indication

The most common drug indication was against hepatitis C virus (HCV) infection (111; 8.43%), followed by porphyria acute (85; 6.46%) and human immunodeficiency virus (HIV) infection (72; 5.47%). Other common conditions included neoplastic diseases, infectious diseases, rheumatic immune system diseases, and psychoneurotic diseases.

#### Drug distribution

**Fig. 3:**
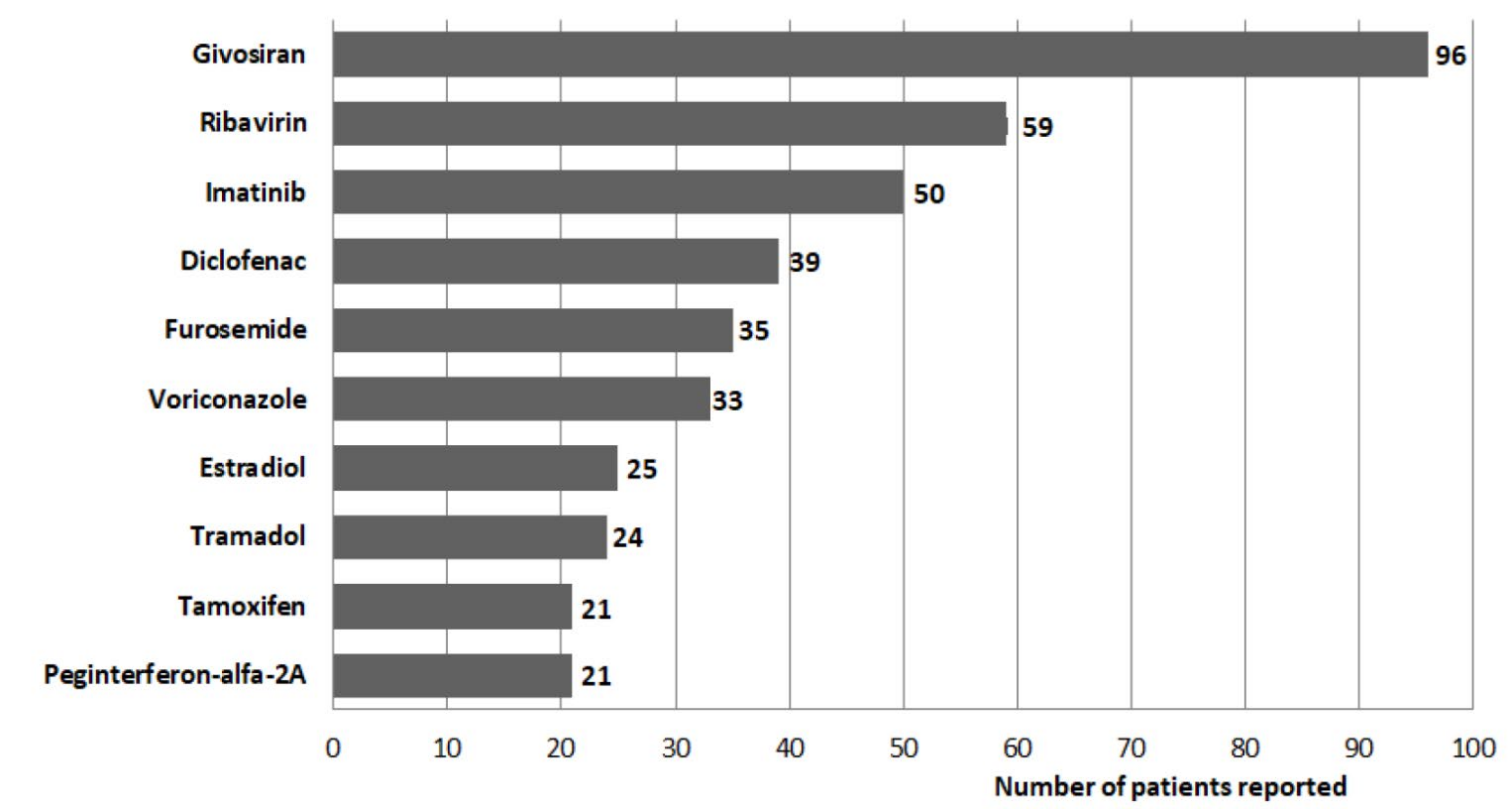
Top ten signaling drugs with the most reports

In the analysis table (Table 2), porphyria-associated drugs were most frequently reported for anti-infective drugs (211; 27.73%), followed by antitumor and immune drugs (120; 15.77%), digestive system drugs (111; 14.59%), nervous system drugs (89; 11.70%), and cardiovascular system drugs (69; 9.06%). The top ten drugs with the highest number of reports are shown in Fig. 3. The 52 drugs with signals are presented using the ATC code classification in Table 2.

**Table 2:** Signaling drugs associated with porphyria from the FAERS

Drug	Porphyrogenicity*	ATC** code	*n*	ROR (95% two-sided ci)	PRR (χ²)	IC (IC025)		EBGM (EBGM05)		
Anti-infective drugs (*n* = 211)										
Antiviral drugs (*n* = 113)										
Ribavirin	PNP	J05AP01	59	31.51 (24.28, 40.9)	31.42 (1668.45)	4.92 (3.79)	30.21 (24.28)			
Efavirenz	PRP	J05AG03	11	8.31 (4.59, 15.04)	8.3 (70.12)	3.04 (1.68)	8.25 (5.02)			
Nevirapine	PRP	J05AG01	9	14.79 (7.68, 28.5)	14.77 (114.85)	3.88 (2.01)	14.69 (8.48)			
Ritonavir	P	J05AE03	9	4.2 (2.18, 8.09)	4.2 (21.8)	2.06 (1.07)	4.18 (2.41)			
Harvoni	NC	/	7	4.18 (1.99, 8.79)	4.18 (16.87)	2.06 (0.98)	4.17 (2.24)			
Truvada	PNP	J05AR03	6	4.19 (1.88, 9.34)	4.19 (14.49)	2.06 (0.92)	4.17 (2.13)			
Kaletra	NC	/	5	10.78 (4.48, 25.95)	10.77 (44.18)	3.42 (1.42)	10.74 (5.15)			
Dolutegravir	PNP	J05AJ03	4	4.75 (1.78, 12.67)	4.75 (11.8)	2.24 (0.84)	4.74 (2.08)			
Atazanavir	PRP	J05AE08	3	7.21 (2.32, 22.4)	7.21 (16.01)	2.85 (0.92)	7.2 (2.79)			
Antibacterial drugs (*n* = 49)										
Ciprofloxacin	PNP	J01MA02	18	5.69 (3.57, 9.06)	5.69 (68.68)	2.49 (1.57)		5.63 (3.82)		
Doxycycline	PNP	J01AA02	13	10.24 (5.93, 17.69)	10.24 (107.39)	3.34 (1.94)		10.15 (6.43)		
Nitrofurantoin	P	J01XE01	9	24.38 (12.65, 46.98)	24.32 (200.05)	4.6 (2.38)		24.18 (13.96)		
Imeth	NC	/	6	6.35 (2.85, 14.17)	6.35 (26.94)	2.66 (1.19)		6.33 (3.23)		
Cephalexin	PNP	/	3	10.18 (3.28, 31.62)	10.17 (24.77)	3.34 (1.08)		10.15 (3.93)		
Antifungal drugs (*n* = 33)										
Voriconazole	PRP	J02AC03	33	33.84 (23.95, 47.81)	33.73 (1024.83)	5.04 (3.57)		33 (24.71)		
Antiparasitic drug (*n* = 10)										
Hydroxychloroquine	PSP	P01BA02	10	7.65 (4.11, 14.25)	7.65 (57.38)	2.93 (1.57)		7.6 (4.52)		
Antituberculosis drugs (*n* = 6)										
Rifampicin	P	J04AB02	6	15.54 (6.96, 34.66)	15.51 (81.15)	3.95 (1.77)		15.45 (7.9)		
Antitumor and immune drugs (*n* = 120)										
Imatinib	PNP	L01EA01	50	10.69 (8.06, 14.17)	10.68 (423.68)	3.37 (2.54)		10.35 (8.17)		
Peginterferon-alpha-2 A	PNP	L03AB11	21	7.93 (5.15, 12.2)	7.93 (125.28)	2.97 (1.93)		7.83 (5.46)		
Docetaxel	PNP	L01CD02	15	3.33 (2, 5.54)	3.33 (24.24)	1.73 (1.04)		3.31 (2.16)		
Interferon Alfa	PNP/PSP	/	13	21.69 (12.56, 37.46)	21.64 (253.74)	4.42 (2.56)		21.46 (13.58)		
Leflunomide	PNP	L04AA03	12	8.54 (4.84, 15.08)	8.54 (79.2)	3.08 (1.75)		8.48 (5.27)		
Betaferon	PNP	L03AB08	6	15.63 (7, 34.86)	15.6 (81.68)	3.96 (1.77)		15.54 (7.94)		
Busulfan	PSP	L01AB01	3	6.02 (1.94, 18.68)	6.01 (12.51)	2.59 (0.83)		6 (2.33)		
Digestive system drugs (*n* = 111)										
Givosiran	NP	A16AX16	96	5139.78 (4011.33, 6585.66)	3506.26 (314573.85)	11.68 (9.11)		3278.43 (2664.32)		
Nitisinone	NC	A16AX04	15	196.81 (117.78, 328.85)	193.18 (2838.88)	7.58 (4.54)		191.22 (124.45)		
Nervous system drugs (*n* = 89)										
Tramadol	PNP	N02AX02	24	9.15 (6.11, 13.69)	9.14 (171.18)	3.17 (2.12)		9.01 (6.43)		
Levetiracetam	PNP	N03AX14	16	3.92 (2.39, 6.41)	3.91 (34.35)	1.96 (1.2)		3.88 (2.57)		
Phenytoin	P	N03AB02	13	9 (5.21, 15.55)	9 (91.61)	3.16 (1.83)		8.93 (5.65)		
Valproic Acid	P	N03AG01	9	4.6 (2.39, 8.87)	4.6 (25.23)	2.2 (1.14)		4.58 (2.65)		
Carbamazepine	P	N03AF01	8	4.36 (2.18, 8.74)	4.36 (20.61)	2.12 (1.06)		4.34 (2.43)		
Diazepam	PNP	N05BA01	7	4.93 (2.34, 10.35)	4.92 (21.79)	2.29 (1.09)		4.91 (2.63)		
Mirtazapine	PNP	N06AX11	6	4.16 (1.87, 9.29)	4.16 (14.36)	2.05 (0.92)		4.15 (2.12)		
Haloperidol	PNP	N05AD01	6	5.96 (2.67, 13.3)	5.96 (24.67)	2.57 (1.15)		5.94 (3.04)		
Cardiovascular system drugs (*n* = 69)										
Furosemide	PNP	C03CA01	35	19.96 (14.27, 27.92)	19.92 (614.19)	4.28 (3.06)		19.47 (14.71)		
Torsemide	NC	/	9	145.9 (75.42, 282.26)	143.89 (1269.43)	7.16 (3.7)		143.02 (82.34)		
Hydralazine	P	C02DB02	7	27.29 (12.98, 57.41)	27.22 (176)	4.76 (2.26)		27.1 (14.55)		
Propafenone	PSP	C01BC03	7	37.87 (18, 79.69)	37.74 (249.17)	5.23 (2.49)		37.56 (20.16)		
Nife	NC	/	6	19.02 (8.53, 42.45)	18.99 (101.86)	4.24 (1.9)		18.92 (9.67)		
Nifedipine	PNP	C08CA05	5	11.09 (4.61, 26.7)	11.08 (45.71)	3.47 (1.44)		11.05 (5.3)		
Urogenital system drugs (*n* = 66)										
Estradiol	PSP	G03CA03	25	6.47 (4.36, 9.61)	6.47 (113.55)	2.67 (1.8)		6.37 (4.58)		
Tamioxifen	NC	/	21	49.52 (32.16, 76.26)	49.29 (979.51)	5.6 (3.64)		48.6 (33.87)		
Progesterone	PRP	G03DA04	9	14.92 (7.75, 28.75)	14.9 (116.03)	3.89 (2.02)		14.82 (8.56)		
Oxybutynin	PNP	G04BD04	7	7.78 (3.7, 16.36)	7.78 (41.16)	2.95 (1.41)		7.75 (4.16)		
Tolterodine	PNP	G04BD07	4	7.9 (2.96, 21.08)	7.89 (24.02)	2.98 (1.12)		7.88 (3.46)		
Dermatological drugs (*n* = 55)										
Diclofenac	PNP	D11AX18	39	6.96 (5.06, 9.56)	6.95 (193.57)	2.76 (2.01)		6.8 (5.21)		
Fluconazole	NC	D01AC15	16	21.08 (12.87, 34.51)	21.04 (302.06)	4.38 (2.67)		20.82 (13.78)		
Hematological systemic drugs (*n* = 32)										
Hemin	NP	B06AB01	15	697.64 (412.59, 1179.62)	653.91 (9680.37)	9.34 (5.52)		647.28 (417.09)		
Erythropoietin	NP	B03XA01	12	10.66 (6.04, 18.82)	10.65 (104.08)	3.4 (1.93)		10.57 (6.57)		
Iron sucrose	NP	/	5	11.01 (4.57, 26.5)	11 (45.29)	3.45 (1.44)		10.96 (5.26)		
Musculoskeletal system drugs (*n* = 8)										
Rocuronium	NP	M03AC09	5	22.51 (9.35, 54.22)	22.46 (102.2)	4.48 (1.86)		22.39 (10.73)		
Pamidronic acid	NP	M05BA03	3	7.93 (2.55, 24.63)	7.93 (18.12)	2.98 (0.96)		7.91 (3.07)		

*Predicted porphyrogenicity of drugs by https://www.drugs-porphyria.org/

**The international drug Anatomical Therapeutic Chemical (ATC) codes were used to classify drugs

*Abbreviations* *p* Porphyrinogenic, *PRP* probably porphyrinogenic, *PSP* possibly porphyrinogenic, *PNP* probably not porphyrinogenic, *NP* not porphyrinogenic, *NC* not yet classified

#### Exploration of porphyrinogen in drugs

The Norwegian Porphyria Centre (NAPOS) has collaborated with the European Porphyria Network (Epnet) and many porphyria experts to create a database (https://www.drugs-porphyria.org/), providing drug safety information on acute porphyria for more than 1,000 drugs, classifying the risk of porphyria from non-porphyritic drugs (which can be used safely) to high-risk porphyritic drugs (which can be used only for emergency indications and under close clinical monitoring) into five levels. We searched the database and divided the signaling drugs into six categories: porphyrinogenic (P), probably porphyrinogenic (PRP), possibly porphyrinogenic (PSP), probably not porphyrinogenic (PNP), not porphyrinogenic (NP), and not yet classified (NC). Among the 52 signaling drugs, 16 were predicted to have a porphyrinogens and thus classified as P, PRP, or PSP, with strong clinical warnings. Twenty-eight drugs were predicted not to have porphyrinogens and were thus classified as NP (six) or PNP (22). The remaining eight drugs were NC (Table 2).

## Discussion

In this study, we collected porphyria AEs from 1470 reporters on the FAERS using four algorithms “ROR”, “PRR,” “BCPNN,” and “MGPS”; 406 drugs were obtained by combining different trade names and generic names representing the same drug, 52 drugs with signals were identified by all four algorithms. Anti-infective, antitumor and immune, digestive system, nervous system, cardiovascular system, urogenital system, dermatological system, hematological system, and musculoskeletal system drugs had high signals. Therefore, extreme caution must be taken to ensure that porphyrinogenic drugs are not prescribed to carriers of porphyria genetic mutations.

Porphyrinogenic drugs are potentially life-threatening to patients with hepatic porphyria and should thus be contraindicated. Early identification and removal of the offending drug, along with immediate treatment, are life-saving [23]. Development of photosensitivity or production of dark urine during drug therapy suggests the possibility of drug-induced PCT, which may respond to cessation of the offending drug [24]. However, these drugs are chemically unique and apparently structurally unrelated, making it difficult to pinpoint the culprit due to conflicting results published in the literature. Thunell et al. [9, 25]. developed a risk assessment model for individual patients receiving a drug, which formed the basis of the www.drugs-porphyria.org database where information on more than 1000 drugs is available for review. Although drug porphyrogenicity prediction can guide drug prescription and reduce drug risk, its accuracy requires verification in clinical practice. The FAERS collects ADR data from the United States and Europe through the MedWatch reporting system [26] and provides an opportunity to perform real-world studies on drug toxicity monitoring [27].

From our analysis, 1470 cases were reported with porphyria AEs in the FAERS, and 406 drugs were obtained by combining the trade names and generic names. The most common patient age was 18–44 years, with more cases involving females than males. Porphyria typically occurs in women between 20 and 30 years of age and 2–4 days prior to menstruation, as ovarian hormones, particularly progesterone, are potent inducers on ALAS1 [28, 29]. Young women are also at high risk for drug-induced porphyria, as shown in this study. Regarding elderly patients, porphyria AEs were frequently reported due to the higher number of drugs prescribed for multiple comorbidities, increasing the risk of encountering porphyrinogenic drugs. Approximately 90% of acute attacks related to AIP occur in women [30]. However, in this study, only 47.07% of cases were reported to have occurred in women. This may be explained in part by male patients carrying porphyria genetic mutations seldomly receiving early diagnosis or drug prescription.

The interval between drug initiation and onset of porphyria as an AE varies greatly, with 1 month (106; 39.70%) as the most common duration. Various drugs have been implicated in exacerbating acute attacks [31], i.e., porphyrinogenic drugs [32]. These drugs deplete the heme pool by inducing or inhibiting cytochrome enzymes (CYP), or abnormally degrading heme [12, 33]. In this study, antiviral drugs were the most common signaling drugs. In a previous study, the association between HIV and HCV infection with PCT was well established [34]. The high dosage of ribavirin could increase hepatic iron levels via hemolysis. The excess of iron in the cytosol of hepatocytes can reduce the action of uroporphyrinogen decarboxylase (UROD) and cause accumulation of its precursor [35]. Moreover, certain antiretroviral drugs also precipitate acute porphyria, such as atazanavir and ritonavir, which inhibit CYP-3A4, leading to heme depletion in hepatocytes, leading to compensatory activation of heme synthesis and toxic accumulation of ALA and PBG precursors in patients who are carriers of acute porphyria genetic mutations [36]. Appropriate antiretroviral regimens should be prescribed with vigilance to these patients. When patients who have been prescribed antiretroviral drugs experience unexplained abdominal pain or skin-photosensitivity symptoms, physicians must consider and closely monitor drug-induced porphyria.

The anti-tuberculosis drug rifampin and anti-fungal drug voriconazole induce or inhibit CYP-450 and provoke a porphyria attack [37]. In 2017, Zaman Babar et al [38]. reported pure motor axonal neuropathy, the peripheral neuropathy of AIP, triggered by anti-tuberculous therapy in an undiagnosed case of acute intermittent porphyria. Most first-line anti-tuberculous drugs are associated with acute attacks of porphyria, its mechanism of action includes: (1) activation of ALAS1 transcription and translation by inducing CYP expression; (2) irreversible inhibition of CYP and compensative activation of heme synthesis; (3) ALAS1 expression induction. Therefore, to prevent the acute onset of latent porphyria, anti-tuberculosis drugs should be used with caution.

Many people develop phototoxicity after using voriconazole [28, 39]. Voriconazole intake is subject to hepatic metabolism by CYP-450 enzymes. Voriconazole serum concentrations maintained between 1.5 and 4 µg/mL are generally safe; however, the possibility of hepatotoxicity cannot be excluded [40], with carriers of porphyria genes being at a greater risk.

Many psychotropic drugs have been implicated in exacerbating acute attacks [41, 42]. However, antipsychotics are often used in acute attacks of porphyria as agents for neuropathic abdominal pain.

This study suggests that immunomodulating agent drugs, like Leflunomide, are associated with porphyria attacks. System lupus erythematosus (SLE) has been associated with porphyria since 1952 [43]. Hydroxychloroquine (HCQ) is often prescribed to patients with SLE to reduce flares; chloroquine and hydroxychloroquine may induce AIP in these patients. Moreover, the use of medium–high doses (250–500 mg/d chloroquine and 200–400 mg/d HCQ) may cause liver toxicity in patients with PCT [44]. However, the mechanism by which immunomodulatory drugs induce porphyria attacks is not well understood. In clinical practice, clinicians should monitor for acute attacks of porphyria when patients using immunomodulators have severe abdominal pain and neuropsychiatric manifestations [24].

Previous studies have reported on the relationship between antitumor drugs and porphyria. Imatinib mesylate is a tyrosine kinase inhibitor [45] that is primarily used to treat chronic myelogenous leukemia (CML). Cutaneous adverse events associated with imatinib are common, while the pathogenesis of pseudoporphyria is unclear. Mahon et al. [45]. speculated that the mechanism of imatinib may be associated with the modulation of c-Kit pathways, disrupting normal melanocyte biology and impairing photoprotective mechanisms. A possible relationship between chemotherapeutic agents and the occurrence of PCT has been discussed in case reports on varying drugs [44]. Manzione et al. speculate that certain chemotherapeutics may induce ALAS1 expression by inhibiting CYP450, which increases heme and porphyrin precursors [46].

Small interfering RNA (givosiran) [47] and hemin are agents without porphyrinogens that are used to stop acute porphyria attacks. Meanwhile, patients who receive givosiran or hemin are at increased risk of disease exacerbation. Consequently, these drugs have been designated as causative agents due to this indication bias when in fact they may not be. Indication of prescription drugs as an error reported as an AE may also occur in this self-reporting system [48]. Interferon (IFN)-α was frequently reported, likely due to its combination with porphyrinogenic antiviral drugs. Such drugs without porphyrinogens must be manually removed from the signaling drugs. Regarding other signal drugs that were predicted as NP, PNP, or NC in the porphyria network drug database, more information is needed to redefine their porphyrinogen status and classification.

This study has certain limitations. First, the FAERS technology does not address all challenges regarding the detection and analysis of adverse drug reaction signs. Hence, the signals from FAERS were used only for qualitative research. Second, false reporting, incomplete reporting, underreporting, and arbitrariness are also included in the data. Third, patients who develop an acute attack may have been simultaneously exposed to multiple drugs and infection or stress, rendering the attribution of blame uncertain. Further research is needed to address these limitations of FAERS.

## Conclusions

Patients who experience drug-induced porphyria generally have bad outcomes. Hence, considerable care must be taken to ensure that carriers of acute porphyria genetic mutations are not prescribed porphyrinogenic drugs. The analysis of FAERS reports provides critical information on drug porphyrogenicity, allowing rational and evidence-based drug prescription and improving the accuracy of predicted porphyrogenicity by model algorithms.

## Data Availability

The data that support the findings of this study are available from the corresponding author upon reasonable request. Some data may not be made available because of privacy or ethical restrictions.

## Abbreviations

ADP: Acid dehydratase deficient porphyria
ADR: Adverse drug reaction
AE: Adverse event
RAHP: Acute hepatic porphyria
AIP: Acute intermittent porphyria
ALA: Aminolevulinic acid
ALAS: Aminolevulinic acid synthase
ATC: Anatomical therapeutic chemical
BCPNN: Bayesian confidence propagation neural network
CEP: Congenital erythropoietic porphyria
CML: Chronic myelogenous leukemia
CYP450: Cytochrome P450
EPP: Erythropoietic protoporphyria
FAERS: FDA’s Adverse Event Reporting System
HCP: Hereditary coproporphyria
HCV: Hepatitis C virus
HCQ: Hydroxychloroquine
HIV: Human immunodeficiency virus
IFN: Interferon
MGPS: Multi-item gamma Poisson shrinker
NAPOS: Norwegian Porphyria Centre
NC: Not yet classified
NP: Not porphyrinogenic
P: Porphyrinogenic
PBG: Porphobilinogen
PCT: Porphyria cutanea tarda
PNP: Probably not porphyrinogenic
PRR: Proportional reporting ratio
PRP: Probably porphyrinogenic
PSP: Possibly porphyrinogenic
ROR: Reporting odds ratio
UROD: Uroporphyrinogen decarboxylase
VP: Variegate porphyria
XLP: X-linked protoporphyria

## References

[1] Anderson KE, Lobo R, Salazar D, et al. Biochemical diagnosis of Acute hepatic Porphyria: updated Expert recommendations for Primary Care Physicians[J]. Am J Med Sci. 2021;362(2):113–21. 33865828 10.1016/j.amjms.2021.03.004

[2] Phillips JD. Heme biosynthesis and the Porphyrias[J]. Mol Genet Metab. 2019;128(3):164–77. 31326287 10.1016/j.ymgme.2019.04.008 PMC7252266

[3] Puy H, Gouya L, Deybach J-C. Porphyrias[J] Lancet. 2010;375(9718):924–37. 20226990 10.1016/S0140-6736(09)61925-5

[4] Stölzel U, Doss MO, Schuppan D. Clinical guide and update on Porphyrias[J]. Gastroenterology. 2019;157(2):365–e3814. 31085196 10.1053/j.gastro.2019.04.050

[5] Chen B, Solis-Villa C, Hakenberg J, et al. Acute Intermittent Porphyria: predicted pathogenicity of *HMBS* variants indicates extremely low penetrance of the autosomal Dominant Disease: HUMAN MUTATION[J]. Hum Mutat. 2016;37(11):1215–22. 27539938 10.1002/humu.23067 PMC5063710

[6] Elder G, Harper P, Badminton M, et al. The incidence of inherited porphyrias in Europe[J]. J Inherit Metab Dis. 2013;36(5):849–57. 23114748 10.1007/s10545-012-9544-4

[7] Bissell DM, Anderson KE, Bonkovsky HL. Porphyria[J]. N Engl J Med. 2017;377(9):862–72. 28854095 10.1056/NEJMra1608634

[8] Wang B, Bonkovsky HL, Lim JK et al. AGA Clinical Practice Update on Diagnosis and Management of Acute Hepatic Porphyrias: Expert Review[J]. Gastroenterology, 2023: S0016508522013567. 10.1053/j.gastro.2022.11.034 PMC10335308 36642627

[9] Ayala F, Santoianni P. Drug-induced cutaneous Porphyria[J]. Clin Dermatol. 1993;11(4):535–9. 7907270 10.1016/0738-081X(93)90162-6

[10] Franciosi EB, Amano SU, Scharf MJ. Porphyria cutanea tarda associated with nitrofurantoin: a unique drug Reaction[J/OL]. Dermatol Ther, 2020, 33(6). 10.1111/dth.14026 32677740

[11] Roveri G, Nascimbeni F, Rocchi E, et al. Drugs and Acute Porphyrias: reasons for a hazardous Relationship[J]. Postgrad Med. 2014;126(7):108–20. 25387219 10.3810/pgm.2014.11.2839

[12] Hift RJ, Thunell S, Brun A. Drugs in porphyria: from observation to a modern algorithm-based system for the prediction of Porphyrogenicity[J]. Volume 132. Pharmacology & Therapeutics; 2011. pp. 158–69. 2. 10.1016/j.pharmthera.2011.06.001 21704073

[13] Rodriguez EM, Staffa JA, Graham DJ. The role of databases in Drug Postmarketing Surveillance[J]. Pharmacoepidemiol Drug Saf. 2001;10(5):407–10. 11802586 10.1002/pds.615

[14] Weiss-Smith S, Deshpande G, Chung S. 等. The FDA Drug Safety Surveillance Program: adverse event reporting Trends[J]. Arch Intern Med. 2011;171(6):591–3. 21444854 10.1001/archinternmed.2011.89

[15] Hauben M. A brief primer on Automated Signal Detection[J]. Annals Pharmacotherapy. 2003;37(7–8):1117–23. 10.1345/aph.1C515 12841826

[16] van Puijenbroek EP, Bate A, Leufkens HGM. A Comparison of Measures of Disproportionality for Signal Detection in spontaneous Reporting systems for adverse drug Reactions[J]. Pharmacoepidemiol Drug Saf. 2002;11(1):3–10. 11998548 10.1002/pds.668

[17] Dumouchel W. Bayesian Data Mining in large frequency tables, with an application to the FDA spontaneous reporting System[J]. Volume 53. The American Statistician; 1999. pp. 177–90. 3.

[18] Szumilas M. Explaining odds Ratios[J]. Journal of the Canadian Academy of Child and Adolescent Psychiatry = Journal De l’Academie Canadienne De Psychiatrie De L’enfant. Et De L’adolescent. 2010;19(3):227–9. PMC2938757 20842279

[19] Norén GN, Bate A, Orre R. Extending the methods used to screen the WHO Drug Safety Database towards Analysis of Complex associations and Improved Accuracy for rare Events[J]. Stat Med. 2006;25(21):3740–57. 16381072 10.1002/sim.2473

[20] Ooba N, Kubota K. Selected control events and Reporting Odds Ratio in Signal Detection Methodology[J]. Pharmacoepidemiol Drug Saf. 2010;19(11):1159–65. 20669233 10.1002/pds.2014

[21] Hauben M, Madigan D, Gerrits CM. The role of Data Mining in Pharmacovigilance[J]. Exp Opin Drug Saf. 2005;4(5):929–48. 10.1517/14740338.4.5.929 16111454

[22] Evans SJ, Waller PC, Davis S. Use of proportional reporting ratios (PRRs) for Signal Generation from spontaneous adverse drug reaction Reports[J]. Pharmacoepidemiol Drug Saf. 2001;10(6):483–6. 11828828 10.1002/pds.677

[23] Szarfman A, Machado SG, O’Neill RT. Use of Screening algorithms and Computer Systems to efficiently signal higher-than-expected combinations of drugs and events in the US FDA’s spontaneous reports Database[J]. Drug Saf. 2002;25(6):381–92. 12071774 10.2165/00002018-200225060-00001

[24] Almenoff JS, Pattishall EN, Gibbs TG, et al. Novel Statistical Tools for Monitoring the Safety of marketed Drugs[J]. Volume 82. Clinical Pharmacology & Therapeutics; 2007. pp. 157–66. 2. 10.1038/sj.clpt.6100258 17538548

[25] Hollingworth S, Kairuz T. Measuring Medicine Use: applying ATC/DDD Methodology to Real-World Data[J]. Pharmacy. 2021;9(1):60. 33802774 10.3390/pharmacy9010060 PMC8006033

[26] Bruce Wang HL, Belkovsky, Joseph K. Lim. AGA clinical practice update on diagnosis and management of Acute hepatic Porphyria: Expert Review. Gastroenterology. 2023 March;164(3):484–91. 10.1053/j.gastro.2022.11.034 PMC10335308 36642627

[27] Fritsch S, Junior MMM, Brenner FM. Increased photosensitivity? Case report of porphyria cutanea tarda associated with systemic lupus Erythematosus[J]. 23223706

[28] Thunell S, Pomp E, Brun A. Guide to drug porphyrogenicity prediction and drug prescription in the acute Porphyrias[J]. Br J Clin Pharmacol. 2007;64(5):668–79. 17578481 10.1111/j.0306-5251.2007.02955.x PMC2203267

[29] Veronin MA, Schumaker RP, Dixit R. The irony of MedWatch and the FAERS database: an Assessment of Data Input errors and potential Consequences[J]. J Pharm Technol. 2020;36(4):164–7. 34752566 10.1177/8755122520928495 PMC7359666

[30] Huang J, Zhang X, Du J et al. Comparing Different Adverse Effects Among Multiple Drugs Using FAERS Data[J]. 2021. PMC8153695 29295353

[31] Aggarwal A, Kulshreshtha B. Catamenial Acute Intermittent Porphyria Managed with GnRH analogues and Estrogen and Progesterone add-back Therapy[J]. J Pediatr Adolesc Gynecol. 2020;33(4):432–4. 32113877 10.1016/j.jpag.2020.02.009

[32] Innala E, Bäckström T, Bixo M, et al. Evaluation of Gonadotropin-releasing hormone agonist treatment for prevention of menstrual-related attacks in acute Porphyria[J]. Acta Obstet Gynecol Scand. 2010;89(1):95–100. 20021268 10.3109/00016340903390729

[33] Hift RJ, Meissner PN. An analysis of 112 acute porphyric attacks in Cape Town, South Africa: evidence that Acute Intermittent Porphyria and Variegate Porphyria Differ in susceptibility and Severity[J]. Medicine. 2005;84(1):48–60. 15643299 10.1097/01.md.0000152454.56435.f3

[34] Thunell S. [The attack of acute porphyria][J]. Lakartidningen, 2016, 113: DXML. 27622758

[35] Smith AG, De Matteis F. Drugs and the hepatic Porphyrias[J]. Clin Haematol. 1980;9(2):399–425. 7398153 10.1016/S0308-2261(21)00183-1

[36] Tephly TR, Hasegawa E, Baron J. Effect of drugs on Heme Synthesis in the Liver[J]. Metab Clin Exp. 1971;20(2):200–14. 5540185 10.1016/0026-0495(71)90092-8

[37] Hernandez GT. Hepatitis C- and HIV-induced porphyria cutanea Tarda[J]. Am J Case Rep. 2014;15:35–40. 24470839 10.12659/AJCR.889955 PMC3901625

[38] Pellicelli AM, Morrone A, Barbieri L, et al. Porphyria cutanea tarda in an HCV-positive liver transplant patient: a case Report[J]. Ann Hepatol. 2012;11(6):951–4. 23109461 10.1016/S1665-2681(19)31424-3

[39] Higgins LS. Tuberculosis and Porphyria[J]. Clinical infectious diseases: an Official publication of the Infectious diseases. Soc Am. 1999;29(3):693–4. 10.1086/598664 10530477

[40] Babar MU, Z, Hakeem H, Khan S. Pure motor axonal neuropathy triggered by antituberculous therapy in an undiagnosed case of acute intermittent Porphyria[J]. BMJ Case Rep, 2017: bcr2016219105. 10.1136/bcr-2016-219105 PMC5372185 28348263

[41] Bernhard S, Lang KK, Ammann RA, et al. Voriconazole-induced phototoxicity in Children[J]. Pediatr Infect Disease J. 2012;31(7):769–71. 22517339 10.1097/INF.0b013e3182566311

[42] Epaulard O, Leccia M-T, Blanche S, et al. Phototoxicity and photocarcinogenesis associated with Voriconazole[J]. Méd Mal Infect. 2011;41(12):639–45. 22055586 10.1016/j.medmal.2011.09.016

[43] Mihăilă R-G. Voriconazole and the Liver[J]. World J Hepatol. 2015;7(13):1828. 26207164 10.4254/wjh.v7.i14.1828 PMC4506940

[44] Burgovne K, Swartz R, Ananth J, Porphyria. Reexamination of Psychiatric Implications[J]. Psychother Psychosom. 1995;64(3–4):121–30. 8657842 10.1159/000289001

[45] Reddy DS. Clinical pharmacokinetic interactions between antiepileptic drugs and hormonal Contraceptives[J]. Expert Rev Clin Pharmacol. 2010;3(2):183–92. 20369030 10.1586/ecp.10.3 PMC2848501

[46] Esteve-Valverde E, Tapiz-Reula A, Ruiz D, et al. Systemic lupus erythematosus and hydroxychloroquine-related acute intermittent Porphyria[J]. Rheumatol Int. 2020;40(5):777–83. 31865445 10.1007/s00296-019-04500-8

[47] Haendchen L, Jordão JM, Haider O, et al. Porfiria cutânea tarda e lúpus eritematoso Sistêmico[J]. An Bras Dermatol. 2011;86(1):173–5. 21437551 10.1590/S0365-05962011000100035

[48] Mahon C, Purvis D, Laughton S, et al. Imatinib Mesylate-Induced Pseudoporphyria in two Children[J]. Pediatr Dermatol. 2014;31(5):603–7. 24920470 10.1111/pde.12380

[49] Manzione NC, Wolkoff AW, Sassa S. Development of porphyria cutanea tarda after treatment with Cyclophosphamide[J]. Gastroenterology. 1988;95(4):1119–22. 3410226 10.1016/0016-5085(88)90191-6

[50] Scott LJ, Givosiran. First Approval[J] Drugs. 2020;80(3):335–9. 32034693 10.1007/s40265-020-01269-0

[51] Faillie J-L. Indication bias or protopathic Bias? [J]. Br J Clin Pharmacol. 2015;80(4):779–80. 26119706 10.1111/bcp.12705 PMC4594717

